# Concussion/mild traumatic brain injury-related chronic pain in males and females: A diagnostic modelling study

**DOI:** 10.1097/MD.0000000000005917

**Published:** 2017-02-17

**Authors:** Tatyana Mollayeva, J. David Cassidy, Colin M. Shapiro, Shirin Mollayeva, Angela Colantonio

**Affiliations:** a Rehabilitation Sciences Institute, Faculty of Medicine; b Department of Occupational Science and Occupational Therapy, University of Toronto; c Toronto Rehabilitation Institute; d Division of Health Care and Outcomes Research, University Health Network; e Division of Epidemiology, Dalla Lana School of Public Health, University of Toronto; f Toronto Western Hospital, University Health Network; g Youthdale Child & Adolescent Sleep Clinic; h Department of Biology, University of Toronto, Mississauga, Ontario, Canada.

**Keywords:** chronic pain, cultural/social model, environmental/behavioral model, perceived states, physical/medical model, physiological and brain injury-related model, psychological model, sex differences

## Abstract

Pain is an unpleasant, complex, and perceived experience that places a significant burden on patients and clinicians. Its severity may be mediated by emotion, attitude, and environmental influences, and pain may be expressed differently in males and females. Traumatic brain injury (TBI) is frequently associated with chronic pain. This diagnostic modeling study examined sex differences in the construct of chronic pain in patients with delayed recovery from concussion/mild traumatic brain injury (mTBI).

Data were collected from standardized questionnaires, neuroimaging records, and comprehensive clinical assessments. Bivariate associations were calculated using the Spearman correlation coefficient or analysis of variance. We established sex-specific stepwise multivariate linear regression models of factors associated with pain.

Of the 94 participants diagnosed with mTBI (the mean age was 45.20 ± 9.94 years; 61.2% were males; the median time since injury was 197 days [interquartile range 139–416]), head/neck, and bodily pain were reported by 93% and 64%, respectively. No sex differences were identified in pain frequencies or severity. Pain was significantly associated with certain socio-demographic, injury-related, behavioral, and clinical variables. In the multivariable regression analysis, several determinants explained 60% of the pain variance in males and 46% in females.

Pain is common in patients with delayed recovery from mTBI and is significantly associated with potentially modifiable clinical and nonclinical variables. Examining the multidimensional construct of pain in concussion/mTBI through a sex lens garners new directions for future longitudinal research on the pain mechanisms involved in postconcussion syndrome.

## Abstract 2

Supplemental Digital Content is available in the text

## 1 Introduction

**Figure 1:**
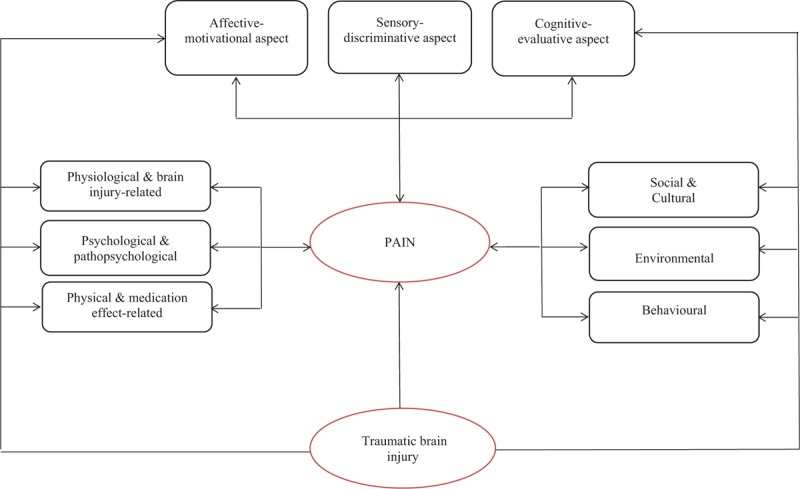
Multidimensional construct of pain in traumatic brain injury. Modified from Price (1999), Brennan, Carr, and Cousins (2007), and Guindon and Hohmann (2009). Unidirectional arrows between constructs (ie, rectangles) and items (ie, ovals) represent reflective models, and from items to constructs, formative models.

Chronic pain is a complex, unpleasant, personal experience that persists after maximal physical healing has been achieved.^[1]^ It places a significant burden on both patients and clinicians.^[2]^ The Stedman Medical Dictionary defines pain as “an unpleasant sensation associated with actual or potential tissue damage, and mediated by specific nerve fibers to the brain where its conscious appreciation may be modified by various factors.”^[3]^ This definition acknowledges that it is possible to experience an injury without experiencing pain and vice versa. Chronic pain may therefore result in unavoidable diagnostic uncertainty.^[4]^ It is also unclear whether a primary complaint of head and/or neck, or bodily pain that persists long after concussion – one of the most common types of mild traumatic brain injury (mTBI) – represents an activation of brainstem structures or a medical problem separate from brain injury mediated by posttraumatic stress disorder (PTSD), hopelessness, disturbed sleep, or depression.^[5–8]^ A systematic review of 23 studies involving 4206 patients with traumatic brain injury (TBI) revealed that, while 51.5% of included patients experienced chronic pain, its frequency in those with mTBIs was twice that in those with more severe injuries, even after adjustment for PTSD.^[7,9,10]^ Latest studies have consistently recorded changes in brain regional connectivity following concussive blows, which may be responsible for the persistent symptomatology observed.^[11,12]^ At the same time, clinicians are cautioned against assuming that pain in TBI is injury-related, and current evidence-based practice suggests that chronic pain in patients with TBI is best assessed holistically, involving an investigation of the patient's medical, physiological, psychological, behavioral, and cognitive-affective strengths and vulnerabilities.^[13,14]^ Research that incorporates this holistic assessment of pain in persons with TBI is still at the conceptual stage, and evidence-based clinical algorithms are currently absent.^[14,15]^ Other challenges include lack of understanding of sex differences in pain expression and perception.^[16–18]^ Decades of neuroendocrine system findings provide a theoretical justification for studying how chronic pain in concussion/mTBI differs between males and females, and this approach is evident in recent international policy statements.^[19,20]^ We researched chronic pain in concussion/mTBI in light of patient sex. We employed Guindon and Hofmann^[21]^ theoretical framework of the multidimensionality of pain, which centers on the theory that pain comprises sensory-discriminative, motivational-effective, and cognitive-evaluative dimensions. To be able to study this framework, we applied a reductionist methodological approach, which postulates that understanding parts (Fig. 1) are important to improve the quality of inductive inferences made regarding the whole.^[20]^ Such a framework provided us with an appropriate context for examining chronic pain in concussion/mTBI; aided in the development of hypotheses; and constituted the basis for our observations, research design, and interpretations. We hypothesized that: chronic pain in males and females with concussion/mTBI is a multidimensional entity, encompassing motivational, cognitive, and sensory dimensions; these dimensions comprise psychological, neurophysiological, cultural, social, and environmental aspects; and the precise elements that constitute chronic pain and their relative importance differ between males and females.

## 2 Methods

### 2.1 Procedure and participants

The Neurology Service of the Toronto Rehab-University Health Network has an exclusive province-wide agreement with the Workers’ Safety and Insurance Board (WSIB) to provide expert diagnostic opinions about persons who have sustained head injuries at work and who continue to experience persistent symptoms when maximal physical healing has occurred. Professionals within psychiatry, neurology, occupational therapy, physiotherapy, and neuropsychology disciplines perform full clinical assessments – including referrals for neuroimaging (ie, structural magnetic resonance imaging [MRI]) and requests for other medical opinions – as deemed necessary at the time of admission. Much of this information (eg, MRI findings, neurocognitive data, and neurological signs) ultimately contributes to a diagnosis for each patient assessed. Most injury referrals are diagnosed as “concussion/mTBI,” where concussion refers to “a complex pathophysiological process affecting the brain, induced by traumatic biomechanical forces.”^[22]^

**Figure 2:**
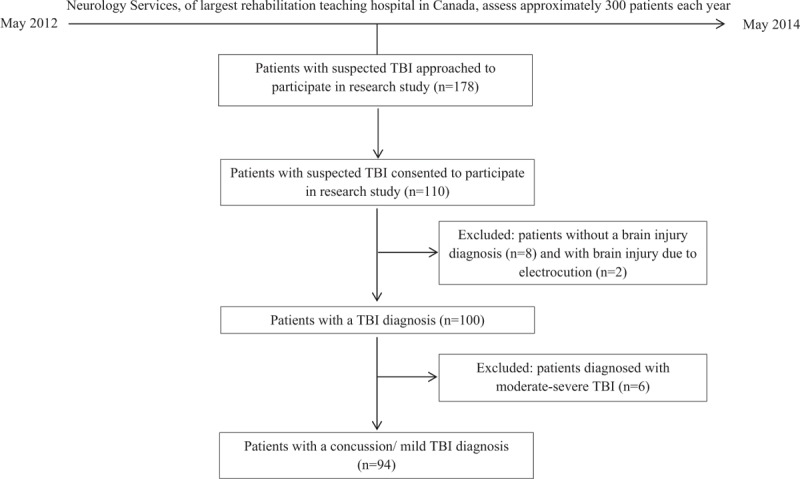
Flow chart depicting process of selection of participating individuals’ data for analysis. TBT = traumatic brain injury.

Injured persons were recruited upon admission to the WSIB clinic at our center between May 2012 and May 2014. Initial contact was made with 178 persons, 110 of whom provided written consent to participate and completed the specified assessments. These participants were asked for written consent to access their clinical and WSIB files, and all gave permission. The researchers were blind to the diagnoses until they were abstracted from the medical chart upon each participant's completion of all assessments. Fourteen percent of the neurology services data were re-abstracted by an independent researcher, and an agreement statistic was obtained to determine concordance between abstraction and re-abstraction. There was excellent agreement (kappa ≥ 0.75) between the 2 abstractors on core variables related to the research questions. To indirectly assess our sample's representativeness, we compared it with a consecutive sample of workers (n = 294) who were referred and assessed in the same clinic during 2003.^[23]^ No significant differences were observed in injury severity, sex, age, or clinical diagnosis. To maintain sample homogeneity in terms of injury severity, we used data for persons (n = 94) with an established diagnosis of concussion/mTBI (Fig. 2).

### 2.2 Instruments and measures

The Pain Visual Analogue Scale (P-VAS) utilized in this study is a patient-reported (PR) measure comprised of pain subscales for current, best, and worst levels of pain.^[24]^ Each Visual Analogue Subscale consists of a 10-cm horizontal line, with endpoints marked as “no pain” and “worst pain ever.” Respondents were instructed to place a mark on the line at the point that best described the current, worst, and best levels of pain intensity experienced over the past 24 hours. Additional PR instruments covered physiological, psychological, cultural, and behavioral variables that were relevant to our hypotheses. Medical files provided data on injury mechanism, presence of loss of consciousness (LOC)/posttraumatic amnesia (PTA), MRI/computed tomography data, and psychosocial status (ie, tension with employer, WSIB, family difficulties, etc.). Medical/clinical data also included the Diagnostic and Statistical Manual of Mental Disorders 4th edition (DSM-IV-TR) diagnoses, comprising mood, anxiety, substance-related, somatoform, and sleep disorders.^[25]^ PTSD falls within the group of anxiety disorders.^[25]^ Measurements by clinicians (ie, all clinical and imaging data) and those obtained directly from participants (ie, all PRs) were collected within a short period from the commencement of investigations, during which no new intervening treatments were commenced. Several items within the PR measures (∼3%) were not completed by participants. We examined missing items and did not observe a relationship with responses on other items within these measures. Therefore, we treated them as missing at random, and used single data imputation to estimate the values of missing items.

We grouped detailed descriptions of the variables under investigation into cultural and social, environmental and behavioral, psychological, physiological and brain-injury related, and physical and medical categories. A detailed description of study variables is presented in Supplementary Table 1.

### 2.3 Statistical analysis

SAS software (version 9.3, SAS Inc., Cary, NC) was used for all data analyses. Means and standard deviations or medians and ranges were used for continuous data, while frequency counts were used for categorical data. The total pain score and its residuals were distributed normally in our sample. We evaluated the normality assumption by examining histograms of all continuous variables and the linearity assumption by examining correlations between continuous variables and scatter diagrams of the dependent variable versus independent variables. We used Spearman correlation coefficients for all a priori defined associations between continuous variables and one-way analyses of variance for categorical/binary explanatory variables to detect collinearity; coefficients between predictor variables >0.7 were considered indicators.^[26]^ We examined residual plots to evaluate error variance assumptions (normality and homogeneity of variance) and examined influence diagnostics (residuals, beta degrees of freedom) to check for outliers. We employed the variance inflation factor (VIF) to assess the inflation of variances of the estimated coefficients; a VIF >6 was considered to distort model estimation.^[27]^ Our final generic and sex-specific regression models were fit with variables detected in individual, rigorously investigated models.

During the developmental stage of our research, we encountered the issue of the potential overlap of the constructs of depression and sleep dysfunction in concussion/mTBI.^[28]^ Our expectation regarding the relationship between insomnia and depression was based on data from the relevant literature: sleep dysfunction is commonly observed in depression, and depression can be the result of poor sleep.^[29–31]^ Moreover, item 3 of the Patient Health Questionnaire-9 (PHQ-9) is Trouble falling asleep or staying asleep or sleeping too much, which is arguably relevant to the construct of insomnia and excessive sleepiness.^[32]^ Therefore, we formulated additional hypotheses about the relationships between depression scores and insomnia scores and conducted an a priori exploration of the collinearity between these constructs.

We applied stepwise multiple linear regressions with elimination^[33]^ to build models of pain (Fig. 1) for each category of variables, grouped by cultural/social, environmental/behavioral, psychological/pathopsychological, neurophysiological/brain-injury related, and physical/medication effect. All hypothesized variables associated with outcomes of interest for the whole group, and for males and females separately, at a statistically significant level of *P* ≤ 0.2 were initially included; all variables identified as significant at *P* ≤ 0.1 were included in final models. Our sample size was adequate to allow accurate estimation of regression coefficients, standard errors, and confidence intervals in all linear regression models.^[34]^ By applying a series of Monte Carlo simulations to examine the impact of the number of subjects per variable (SPV) on the accuracy of estimated regression coefficients and standard errors, on the empirical coverage of estimated confidence intervals, and on the accuracy of the estimated *R*^2^ of the fitted linear regression model, Austin and Steyerberg found that linear regression models require only 2 SPV.^[34]^ We ensured a higher number of SPV in all our models. The Research Ethics Boards at the Toronto Rehab-University Health Network and the University of Toronto approved the protocol of the present study. We followed the Transparent Reporting of a Multivariable Prediction Model for Individual Prognosis or Diagnosis guidelines in the reporting of our results.^[35]^

## 3 Results

Tables 1 and 2 present the characteristics of the 94 participants (45.20 ± 9.94 years; 58 males, 36 females) with concussion/mTBI. The median time since injury was 197 days [interquartile range 139–416]. The major mechanisms of injury were being struck by/against an object or crushed by an object (40%), falls from the same level (17.5%), motor vehicle incidents (13.8%), and being struck by another person (11.2%). Among persons with documented LOC and/or PTA, 31% had experienced LOC and 25% PTA. Among persons who underwent structural MRI imaging, non-specific scattered foci of hyper-intensity were detected in 32%. DSM-IV-TR disorders, including cognitive, adjustment, anxiety, mood, somatoform, substance-related, and sleep disorders were diagnosed in 63%, 51%, 45%, 42%, 29%, 15%, and 10% of our participants, respectively. Fourteen (16%) participants were diagnosed with possible/probable malingering.

**Table 1:**
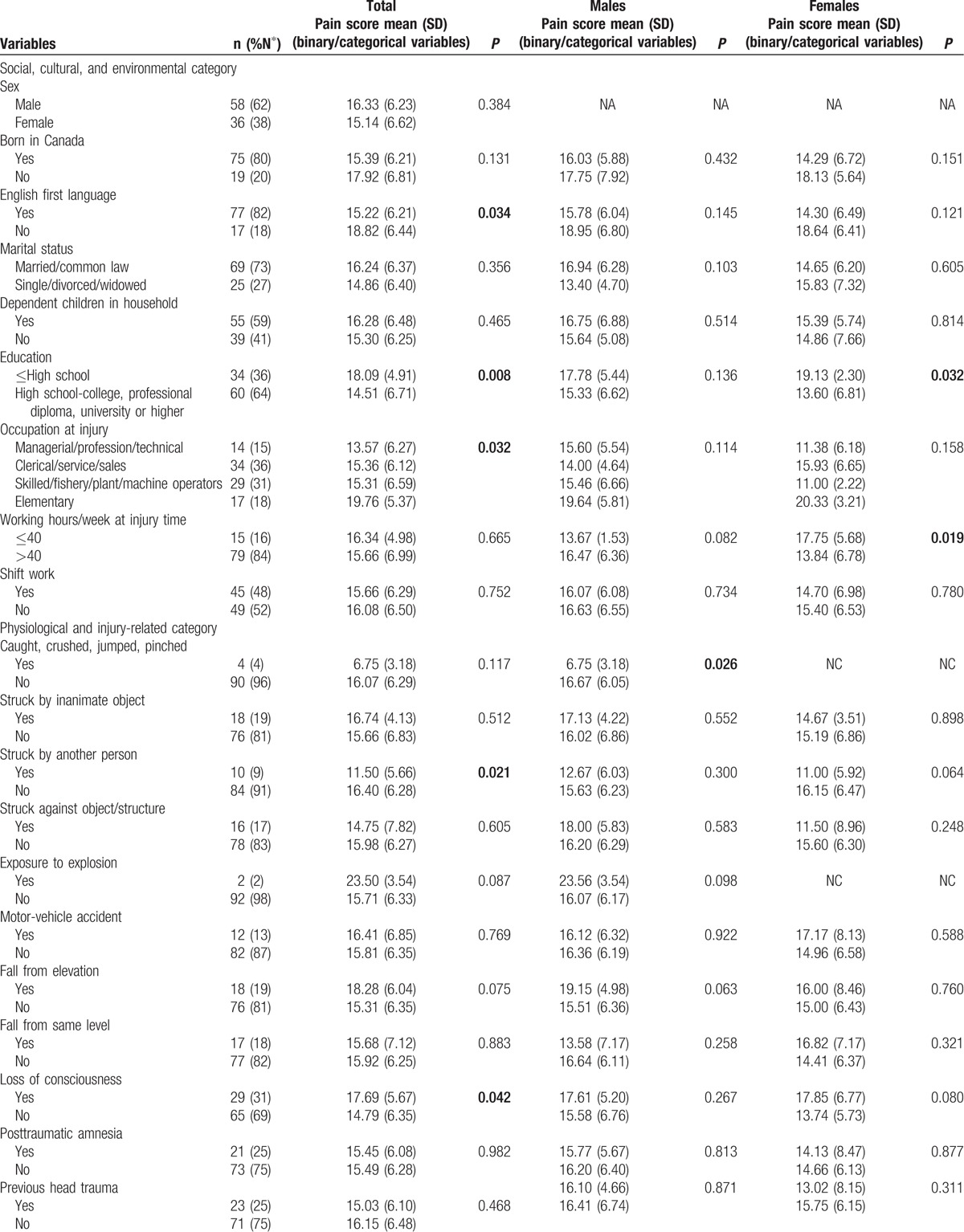
Characteristics of the study population, by sex, and corresponding pain scores.

**Table 1 (Continued):**
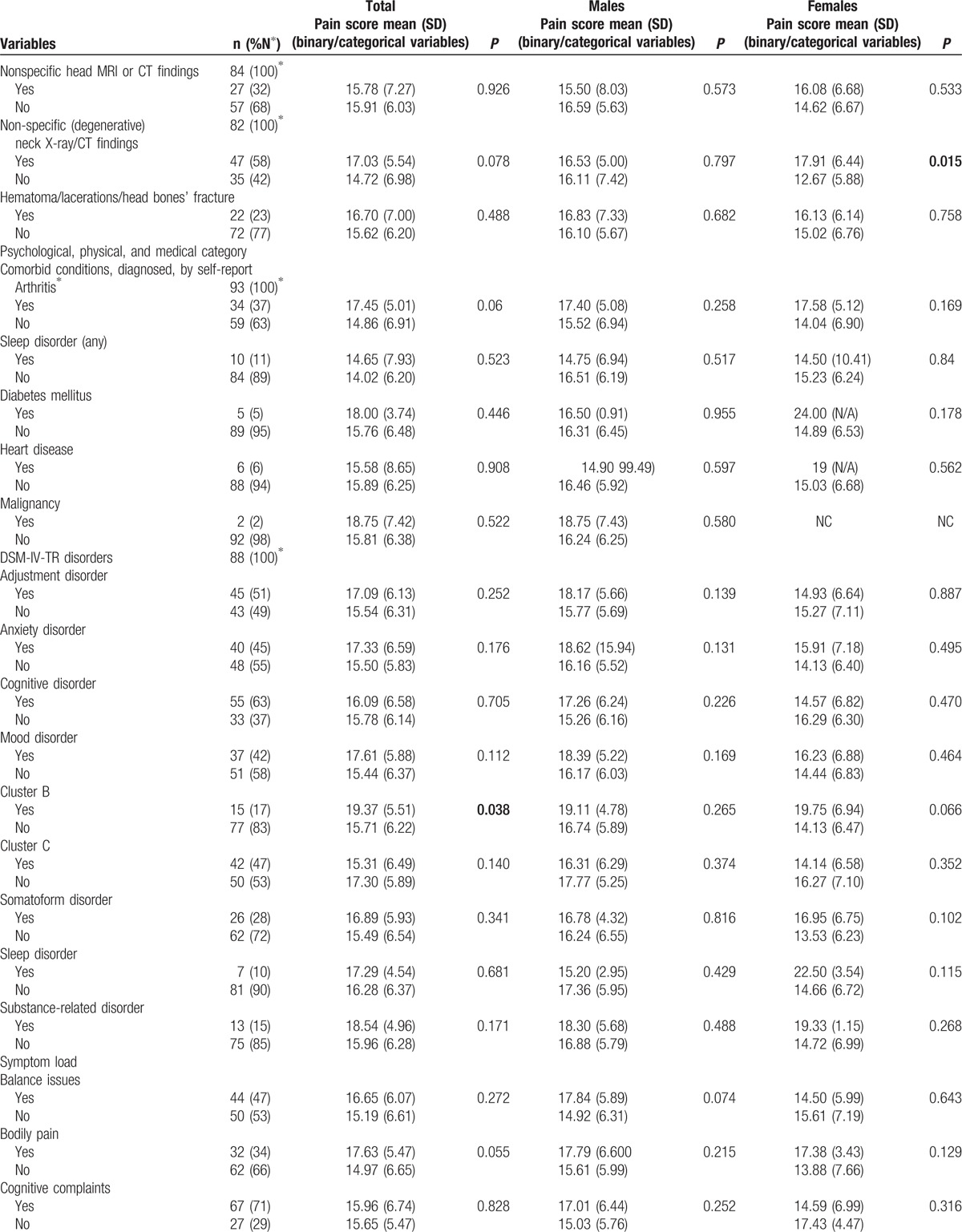
Characteristics of the study population, by sex, and corresponding pain scores.

**Table 1 (Continued):**
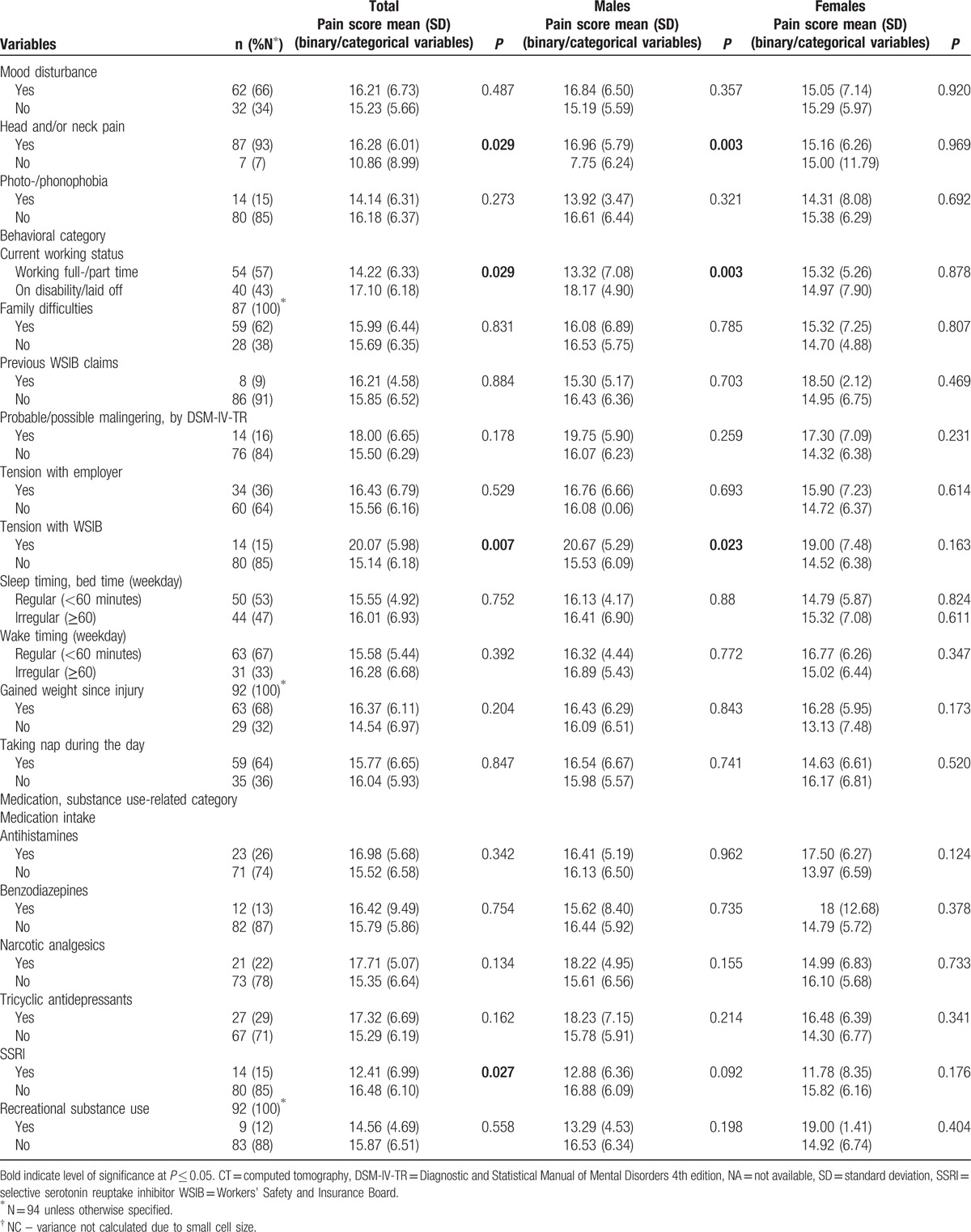
Characteristics of the study population, by sex, and corresponding pain scores.

**Table 2:**
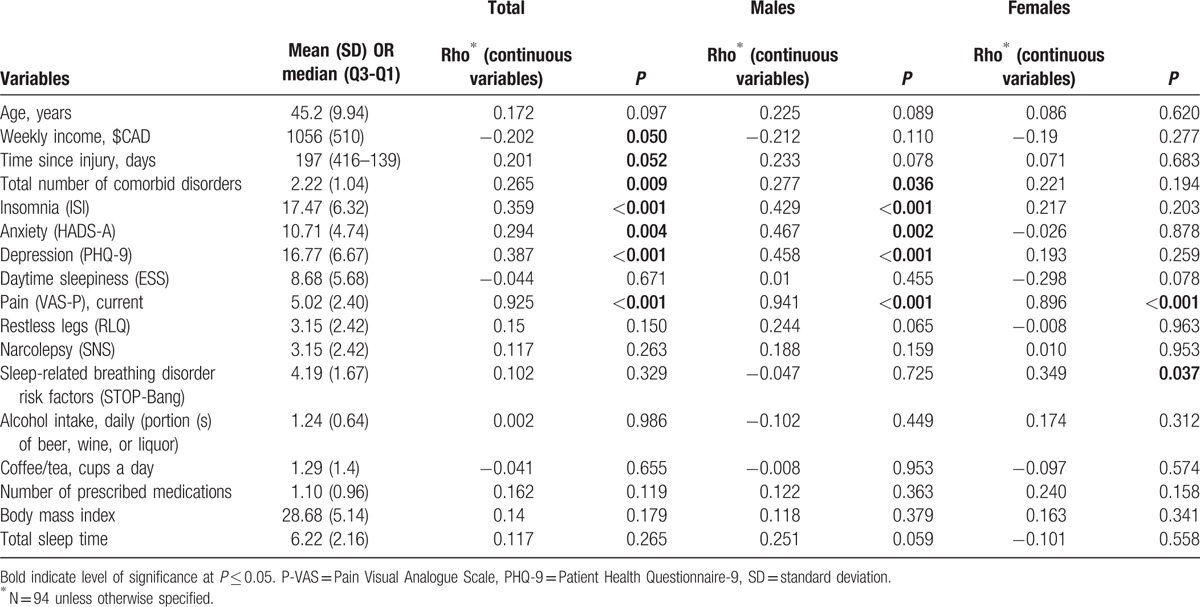
Characteristics of the study population, by sex, and corresponding pain scores for continuous variables.

## 4 Distribution of pain severity by sex and statistical properties of the P-VAS

The mean total P-VAS score for all participants was 15.87 ± 6.37; 16.33 ± 6.23 for males and 15.14 ± 6.62 for females (where 0 and 30 indicate no pain and worst possible pain, respectively). The mean current pain intensity recorded on the P-VAS was 5.02 ± 2.40, with 5.08 ± 2.47 for males and 4.92 ± 2.32 for females (where 0 and 10 indicate no pain and worst possible pain, respectively). The mean best pain intensity was 3.42 ± 2.30, with 3.61 ± 2.31 for males and 3.11 ± 2.22 for females. Finally, the mean worst pain intensity was 7.43 ± 2.43, with 7.64 ± 2.27 for males and 7.10 ± 2.67 for females. The item–item correlation coefficient (ie, the level of association between the subscales in the measure) ranged from 0.60 for best and worst levels of pain (#2 and #3) to 0.76 for current and worst levels of pain (#1 and #3). Correlation between current and best levels of pain (#1 and #2) within the past 24 hours was 0.72. The 3-item P-VAS did not record subscales that do not correlate with one another (ie, <0.20) or ones that are highly correlated (>0.90). Therefore, we used the total level of pain (sum of current, best, and worst) for more sensitive analyses of the data. The internal consistency of the P-VAS total level of pain, measured by Cronbach α, was 0.87 (ie, current level of pain was 0.75, worst level of pain was 0.84, and best level of pain was 0.87).

### 4.1 Analyses

We began our analyses with a simple correlative approach to test the hypothesized relationships within each domain of the theoretical framework of chronic pain in concussion/mTBI (Fig. 1).^[36]^ We then considered the nature of the relationship between variables at different levels of generality and chronic pain and applied multivariate techniques,^[37]^ aggregating variables associated with chronic pain into simpler organizing themes (domains) (see Fig. 1) to identify covariates of pain that are over-represented among males and females with concussion/mTBI. The final multivariate model was derived using variables that were associated with chronic pain within the simpler organizing domains.

### 4.2 Bivariate analyses

Participants with education to high school completion or less had significantly higher pain scores that those with greater than high school education (18.09 ± 4.91 vs 14.51 ± 6.71, *P* = 0.008). Persons whose injuries resulted from being struck by another person had significantly lower P-VAS total scores than those whose injuries resulted from other causes (11.50 ± 5.66 vs 16.40 ± 6.28, *P* = .021). Participants who experienced LOC during the concussive event and those who reported head and neck pain had significantly higher pain scores than those who did not experience LOC and those without head and neck pain (17.69 ± 5.67 vs 14.79 ± 6.35, *P* = 0.042 and 16.28 ± 6.01 vs 10.86 ± 8.99, *P* = 0.029, respectively). There were significant differences in pain scores between persons who did and did not experience tension with insurer (20.07 ± 5.98 vs 15.14 ± 6.18, *P* = 0.007) and between those who snored during sleep and those who did not (16.44 ± 6.33 vs 13.35 ± 6.19, *P* = 0.051). Participants who were taking selective serotonin reuptake inhibitors (SSRI) at the time of assessment had significantly lower pain scores than who did not (12.41 ± 6.99 vs 16.48 ± 6.10, *P* = 0.027). Spearman correlation coefficients were as follows: P-VAS total scores were positively correlated with insomnia (*r* *=* 0.359, *P* < 0.001), depression (*r* = 0.387, *P* < 0.001), and anxiety (*r* = 0.294, *P* = 0.004) and negatively associated with weekly income (*r* = −0.202, *P* = 0.050). There were no significant effects among the other independent variables.

### 4.3 Bivariate analyses of data from male participants

Male participants who sustained concussion/mTBI by being caught, crushed, pinched, or by jumping by/between object(s) had significantly lower pain total scores (6.75 ± 3.18 vs 16.67 ± 6.05, *P* = 0.026) than those injured in other ways. Pain scores differed significantly between persons who did and did not report head and neck pain at the time of assessment (16.96 ± 5.79 vs 7.75 ± 6.24, *P* = 0.003). Persons with tension with the insurer had significantly higher P-VAS scores (20.67 ± 5.29 vs 15.53 ± 6.09, *P* = 0.023), and those working at the time of assessment had significantly lower scores (13.32 ± 7.08 vs 18.17 ± 4.90, *P* = 0.003). Spearman correlation coefficients showed P-VAS scores were negatively associated with the total number of comorbid disorders (*r* = 0.277, *P* = 0.036), and severity of insomnia, depression (*r* = 0.429 and 0.458, *P* < 0.001, respectively), and anxiety (*r* = 0.467, *P* = 0.002). There were no significant effects among the other independent variables.

### 4.4 Bivariate analyses of data from female participants

Female participants with education levels greater than high school completion had significantly lower P-VAS total scores than others (13.60 ± 6.81 vs 19.13 ± 3.30, *P* = 0.032). P-VAS scores differed significantly between persons who had degenerative changes in their cervical spine (identified by computed tomography or MRI scan) and those who did not (17.92 ± 6.44 vs 12.67 ± 5.88, *P* = 0.015) and between those who snored while sleeping and those who did not (16.70 ± 5.97 vs 10.64 ± 6.05, *P* = 0.009). The P-VAS total score positively correlated with the STOP-Bang (a measure of sleep apnea) total score (*r* = 0.349, *P* = 0.037). No other significant correlations were observed.

The complete ANOVA results for binary and categorical variables and the Spearman correlation coefficients for continuous variables and their associated values with P-VAS, for the total sample and by sex, are given in Tables 1 and 2.

### 4.5 Multivariable regression analyses

**Figure 3:**
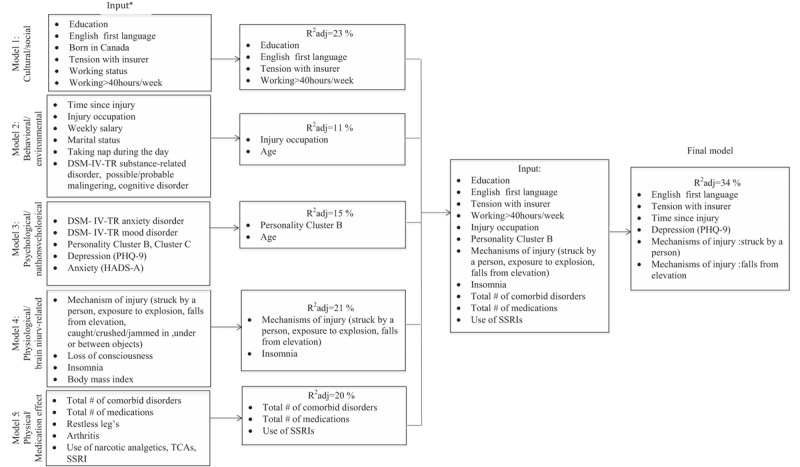
Flow diagram depicting the stepwise multiple regression analysis of pain in males and females combined. ∗Each model age- and sex-adjusted. DSM-IV-TR = Diagnostic and Statistical Manual of Mental Disorders 4th edition, HADS-A = Hospital Anxiety and Depression Scale-Anxiety Subscale, PHQ-9 = Patient Health Questionnaire-9, SSRI = selective serotonin reuptake inhibitor, TCA = tricyclic antidepressant.

**Figure 4:**
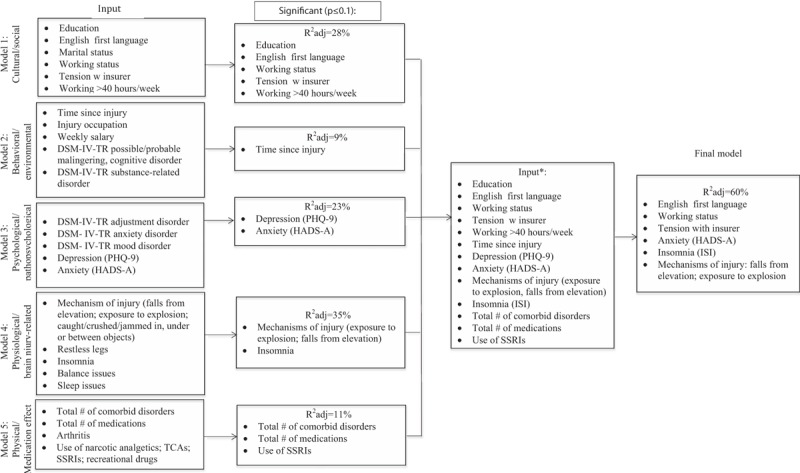
Flow diagram depicting the stepwise multiple regression analysis of pain in males. ∗Each model age-adjusted. DSM-IV-TR = Diagnostic and Statistical Manual of Mental Disorders 4th edition, PHQ-9 = Patient Health Questionnaire-9, SSRI = selective serotonin reuptake inhibitor.

**Figure 5:**
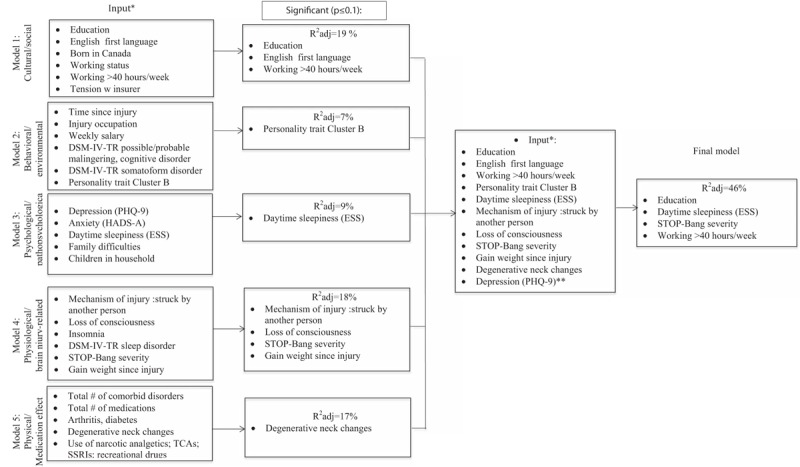
Flow diagram depicting the stepwise multiple regression analysis of pain in females. ∗Each model age-adjusted, ∗∗included due to consistently reported associations. DSM-IV-TR = Diagnostic and Statistical Manual of Mental Disorders 4th edition, PHQ-9 = Patient Health Questionnaire-9, SSRI = selective serotonin reuptake inhibitor.

We fitted our stepwise regression general models and sex-specific models on the bases of the bivariate analyses, associations reported in the literature, and predefined hypotheses (Figs. 3–5). All of our general models were adjusted for age and sex, and each sex-specific model was adjusted for age. Individual models were adjusted for DSM-IV-TR diagnoses of possible/probable malingering and cognitive disorder. We did not observe VIF > 4 for any covariate, suggesting that collinearity did not contribute to the change in regression estimates. We observed, in line with our hypotheses, significant correlations between measures of depression and insomnia (*r* = 0.559, *P* < 0.001). To ensure that this correlation was not driven by overlapping symptoms in the measures of these constructs (ie, PHQ-9 Item 3: Trouble falling asleep or staying asleep or sleeping too much), we conducted our analyses with and without the PHQ-9 sleep item. This resulted in minor changes in the effect of depression and insomnia on pain in the general and final models in males. No changes to the effect of any variables on pain were observed in the final model of pain in females. We report our results without modifying the PHQ-9 measure to be consistent with the previous literature but refer the reader to Supplementary Table 2 for the results of the final regression analyses with the sleep item removed.

The final general regression model for pain tested covariates identified in the earlier general models at *P* ≤ 0.1. After stepwise selection and using a threshold level of significance of *P* ≤ 0.05, the final fully adjusted model of pain including age and sex explained 34.5% of the variance and contained 6 independent variables. These are English as the 1st language (β = −3.327, *P* = 0.026), tension with insurer (β = 4.187, *P* = 0.022), depression (β = 0.385, *P* < 0.001), mechanism of injury being struck by another person (β = −3.698, *P* = 0.007), mechanism of injury a fall from a height (β = 3.157, *P* = 0.020), and time since injury (β = 0.0009, *P* = 0.056). The final fully adjusted model of pain in males explained 60% of the variance and contained 7 variables. These are English as the 1st language (β = −3.160, *P* = 0.030), mechanism of injury exposure to explosion (β = 7.011, P = 0.020), mechanism of injury a fall from a height (β = 3.233, *P* = 0.023), anxiety (β = 0.469, *P* < 0.001), insomnia (β = 0.346, *P* < 0.001), working status (β = −3.547, *P* = 0.004), and tension with insurer (β = 3.288, *P* = 0.035). The final fully adjusted model of pain in females explained 46.2% of the variance and contained 4 variables. These are education level greater than high school completion (β = −4.405, *P* = 0.039), STOP-Bang severity (β = 1.750, *P* = 0.006), daytime sleepiness (β = −0.50, *P* = 0.004), and working more than 40 hours per week at the time of injury (β = −4.457, *P* = 0.019) (Table 3).

**Table 3:**
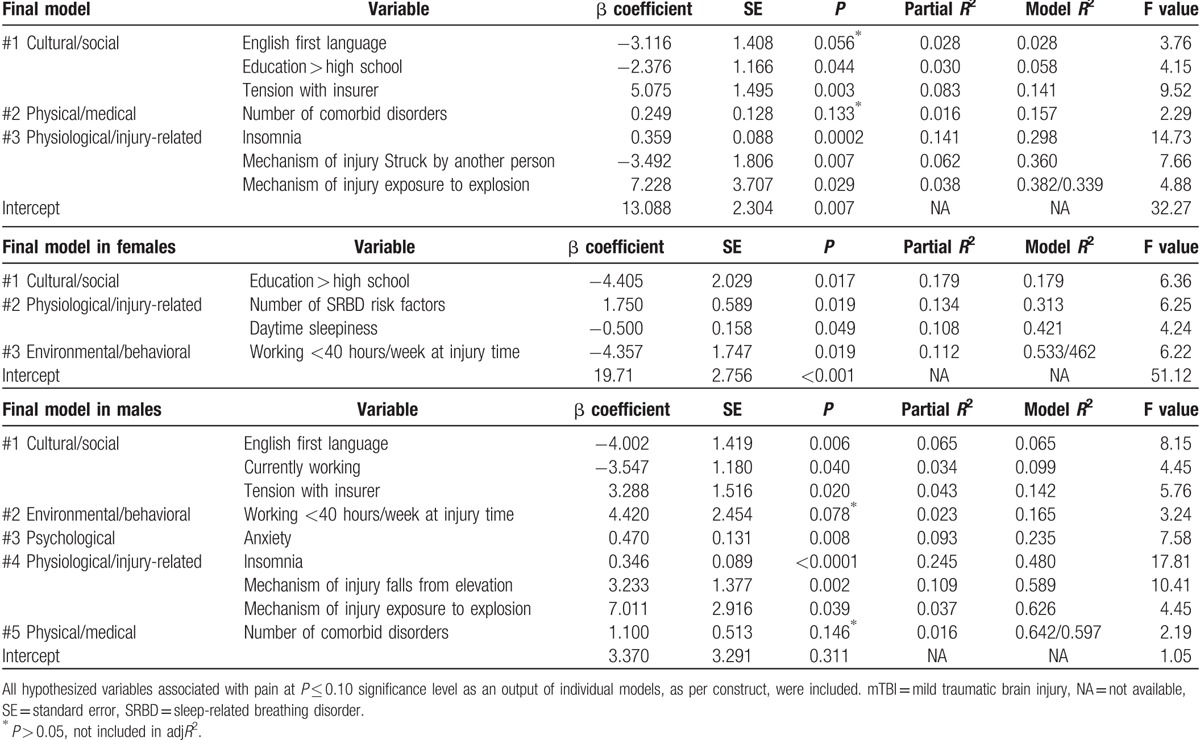
Summary of the stepwise multiple regression analysis for the final models of pain in males and females with mTBI combined, and stratified by sex.

## 5 Discussion

In this diagnostic modeling study of middle-aged (45.20 ± 9.94 years) persons with delayed recovery from concussion/mTBI, we demonstrate that head/neck and bodily pain are highly pervasive, being present in 93% and 64% of our sample, respectively, with no sex-differences identified in pain frequencies. These numbers are significantly higher than reported by Martelli et al^[7]^ who studied persons 6 months postinjury for head and neck pain (44%), and notably higher than reported in a systematic review of chronic pain in TBI (50%).^[9]^ Sex stratification of our results provided a more precise view of the factors associated with chronic pain across domains, and revealed that pain intensity/unpleasantness appears to be construed differently by males and females with concussion/mTBI, and that only sex-specific analyses allowed us to capture these differences.

### 5.1 Pain in males

Our multivariate analyses of chronic pain in males outlined depression and anxiety as most significant covariates, alone explaining 31% of variance in chronic pain; with depression forced out of the model when insomnia entered. Our results are consistent with previous discoveries on the topic of chronic pain, suggesting that the emotional state of mind (or situation or experience) is related to the perception of pain,^[38–40]^ and that poor sleep may be a mediator in the relationship between depression and pain. Our results go along with the concept of pain proposed by the ancient Greeks, who considered chronic pain to be the opposite of pleasure, viewing it as an emotion.^[1,41]^ Research in the general population noted that that the frequency of symptoms in any given day was predicted by whether a person slept less than 6 hours or more than 9 hours, with pain perception and modulation being altered in relation to sleep state.^[42]^ Although in general, sensory information is filtered during sleep, relevant inputs threatening body homeostasis may trigger arousal (ie, a rapid return of consciousness),^[43]^ causing sleep fragmentation and frequent awakenings, with patients often reporting insomnia and greater pain upon awakening. Etiologically, such factors as baseline sleep state should be investigated in future research regarding pain and emotional states associated with mTBI. Regardless of cause-effect, our results raise new hypotheses about the potential link between sleep, depression, and pain in mTBI, supporting the compelling evidence that poor sleep may compromise mood and modulate pain.^[42,43]^ Future neuroimaging studies hold significant promise for identifying networks implicated in the affective component of chronic pain in persons with concussion/mTBI.

Chronic pain intensity/unpleasantness appears to be dependent on the mechanism of injury. Individuals injured in explosions and those who fell from heights perceived more severe pain than others did. This supports the findings of a military study that reported that those with blast injuries were more likely to have continuous pain at 2 years postinjury than those who sustained injuries from nonblast causes.^[44]^ Injuries from explosive devices and mines caused slightly over 2% of TBIs in our civilian population, yet they were significantly associated with chronic pain in bivariate analyses and regression models at different levels of aggregation. Our results should be interpreted with caution, given the small number of persons involved; nevertheless, they lend support to the notion that blast injuries may create “a poly-trauma clinical triad,”^[45]^ where chronic pain and persistent postconcussion symptoms are manifestations of more complex wounds to the brain tissue than those sustained by other injury mechanisms. Other researchers proposed that persistent postconcussive symptoms experienced in returning veterans are not brain injury-related, but are manifestations of PTSD.^[46]^ We did not observe the presence of anxiety disorders according to DSM-IV criteria (PTSD, panic disorder, generalized anxiety disorder, etc.) to be associated with chronic pain in either males or females in our sample. On the contrary, another mechanism of injury, falls from heights, which is more likely to result in diffuse TBI because of deceleration forces in the brain when the head hits the ground,^[47]^ appeared to be associated with chronic pain at different levels of aggregation. Our results may support the notion that chronic pain in concussion/mTBI is part of a complex neuropathological process that occurs following brain injury. Therefore, future studies of pain in concussion should continue to integrate injury mechanisms into their hypotheses.^[48]^

English as a first language was associated with less severe pain in males but not in females. Earlier research has acknowledged cultural and sociodemographic factors as important contributors to chronic pain and disability.^[49–51]^ Our bivariate analyses identified an association between pain and occupation; laborers experience more significant pain than other occupations, although this relationship disappears when sociodemographic and other occupational variables are incorporated into the model. This observation may point to the types of duties non-English participants carry out at work.^[52]^ The potential cultural and sociodemographic dimorphism revealed in our study should be further explored. This is particularly relevant in major urban centers in Canada, given its linguistic diversity and growing prevalence of newcomers entering the workforce with languages other than English.^[53,54]^

### 5.2 Pain in females

Our results highlight independent associations between levels of education and chronic pain in females and males in the bivariate analyses. This suggests that chronic pain in concussion/mTBI shares similar mechanisms with chronic pain in other populations.^[55,56]^ The association between level of education and chronic pain was observed at all levels of aggregation in females; in the final model, it accounted for 18% of the observed variance. Previous studies have proposed that lower education is a risk factor for chronic low back pain because those with less education are more likely to be engaged in more physically demanding physical work.^[57]^ In contrast, inconsistent findings were observed about chronic headaches: some researchers reported that females with a higher educational level have more severe headaches, and others reported the opposite.^[58,59]^ Further investigation of sex-related differences in education as it relates to chronic pain in the mTBI population is warranted.

Working >40 hours per week at the time of injury was associated with lower pain severity in females (β = −3.357, *P* = 0.019), but higher pain severity in males (β = 4.420, *P* = 0.078). It is difficult to explain such a difference between the males and females in our sample. One possible explanation is different physical demands placed on males and females. For example, earlier research has found over-activity to be a legitimate part of the construct of chronic pain.^[60]^ With the knowledge that females use socially guided decision making in conditions of uncertainty (such as chronic pain) more often than males,^[61]^ females in our sample may have more accurately attributed pain exacerbation to over-activity than males, and therefore, avoided such activity postinjury. Studies on sex differences in the history of over-activity and chronic pain in concussion/mTBI, starting at the injury event and followed over time, are therefore critical.

Sex as a risk factor for specific sleep disorders in mTBI has gained significant recent attention in the clinical and research communities.^[62,63]^ It has been reported that obstructive sleep apnea, a form of sleep-related breathing disorder (SRBD), remains largely undiagnosed in females, due to variations in clinical presentation (ie, SRBD in females manifests with symptoms of depressive mood, insomnia, morning headaches vs snoring and witnessed apnea in males) as well as higher tolerance to symptoms.^[64]^ At the same time, sleep architecture is more severely affected by SRBD in females compared to males.^[65]^ Our results align with earlier research, emphasizing sleep-related variables (ie, SRBD, daytime sleepiness) as key covariates of chronic pain in females, but not in males. These sex-specific results may indicate more profound sensory-motor integration in upper airway and craniofacial muscles as a result of respiratory instability during sleep in mid-aged females compared to males.^[66]^ At the same time, sensory and motor processes in sleep are reported to be differentially affected by prevailing behavioral states, which are hormone-dependent.^[67]^ The extent and mechanisms by which SRBD influences chronic pain in males and females with concussion/mTBI, and vice versa, and how abnormalities in respiratory and motor control during sleep or behavioral states underlie the relationship between chronic pain and SRBD in males and females, require longitudinal investigation.^[68]^ Our results suggest that future longitudinal studies on chronic pain in concussion/mTBI, a prerequisite for developing treatment interventions, should be sex-specific, given that sex differences have also been detected in circadian clock genes, respiratory control, and stress responses.^[67–69]^

Likewise, daytime sleepiness was an independent covariate of chronic pain in females, but not in males. The mean sleepiness score (Epworth Sleepiness Scale [ESS]) for the study cohort was elevated for both sexes compared with the general population (8.67 ± 5.66, with 8.61 ± 5.70 for males and 8.89 ± 5.70 for females; vs 6.3 ± 3.5 and 5.9 ± 2.2, respectively).^[70]^ In patients with TBI, central nervous system pathology with hypocretin/orexin deficiency is thought to underlie daytime sleepiness.^[71]^ We had no narcolepsy cases in our sample, as determined by the Swiss Narcolepsy Scale and clinical examination. In all cases of excessive sleepiness, our participants had elevated scores on the STOP-Bang measure, similar to those reported in larger population-based studies in patients with a respiratory disturbance index (RDI) of 15 to 30.^[72,73]^ Our results, however, identified a relationship between sleepiness and pain severity independent of SRBD. This may be related to our chosen measure of sleepiness (ie, ESS), which reflects sleepiness in light of social contexts (watching TV, sitting quietly after a lunch without alcohol, sitting during a meeting, etc.), the frequencies of which differ between sexes. Given that perceptions are mostly shaped by personal experience, chronic pain is expected to be differently perceived in males and females. Daytime sleepiness and chronic pain in concussion/mTBI should be interpreted in the context of the patient's overall clinical, social, and cultural profile, taking into account the measure utilized.

### 5.3 Strengths and limitations

A strength of this study is its interdisciplinary approach, crossing biological, psychological, behavioral, and social domains to create a complex context-dependent construct of pain, allowing the identification of associations on multiple levels and improving the quality of inductive inferences. The benefit of this approach lies in identifying previously undescribed associations for further study. Multivariate techniques allow constructs to be aggregated to more functionally meaningful entities. We formulated hypotheses while developing the study that were based on a currently accepted framework of chronic pain multidimensionality. The diagnoses of concussion/mTBI were made by a team of clinicians trained in neurology, psychiatry, psychology, and other relevant disciplines. To the best of our knowledge, there is no earlier integrative study that has examined biological, psychological, behavioral, and social covariates of chronic pain.

Study recruitment was not random; all participants had been injured at work and had jobs to which they could return, though they continued to experience symptoms that interfered with their functioning. We had access to structural imaging data, all of the participants’ medical assessments, and their medical histories, in addition to the data that was directly relevant to this research. Although we had reasonably high response rates, many potential research participants were not enrolled due to lack of informed consent. We retrospectively examined the charts of the consecutive list of participants assessed in the same clinic in 2003 to ensure our sample was representative. Nevertheless, there may have been selection bias toward those with more significant distress, those with less significant physical or cognitive impairments, or those who wanted to understand the cause of their on-going difficulties.

We have used stepwise multiple regressions in building our models and not a hierarchical approach, which many researchers see as a preferable statistical methodology. In light of the number of a priori-hypothesized variables potentially associated with our outcome of interest, the unknown hierarchy between variables in the construct of chronic pain – and due to the fact that only the stepwise multiple regressions allowed us to evaluate the order of importance of variables in the individual modeling process – our research team collectively agreed that, despite the potential limitations (ie, the fit may appear to be better than it is, model simplification, etc.), stepwise multiple regression was the most suitable approach.

We used a Visual Analogue Scale for pain assessment.^[74]^ This method is particularly valuable because it captures the unpleasantness of the pain experience, is sensitive to interventions that alter pain experience, and correlates well with other numeric and verbal rating scales.^[75]^ It has been reported, however, that the visual analogue scale primarily measures pain affect and may not adequately capture the complexity of pain phenomena, particularly sensory and overall intensity domains.^[76]^ We attempted to mitigate this by capturing the sensory location of pain, namely head, neck, or bodily pain. Our statistical analyses used continuous numeric values of the intensity of pain, encompassing current, best, and worst pain. We sought to distinguish covariates of perceived pain by collecting a variety of data related to the hypothesized relationships, using standardized scales and paying attention to specific items within them. Nevertheless, it remains unclear how well the P-VAS reflects the multidimensionality of the construct under study. We evaluated the internal consistency of the measure, and the results indicated that its use was appropriate. Nonresponse bias did not occur. Self-validity of PR outcome data were ensured in our bivariate and multivariate modeling analyses by controlling for intentional production of false exaggerated physical or psychological symptoms (ie, DSM-IV-TR diagnoses of malingering) and cognitive disorder, as determined by the neurology services team. The present study highlights factors associated with the perceptual state of pain at the moment of investigation (a cross-sectional relationship), though the longitudinal relationships remain to be determined. Finally, we explored the construct of chronic pain through biological (ie, sex) lens; factors associated with gender (socialized toughness in men, etc.) may also be involved, but were not explored.

## 6 Conclusions

The results of our study confirm that various hypothesized factors are related to the perception of pain in the chronic phase after concussion/mTBI. The analyses across simple organizing themes provide unique opportunities for future longitudinal research that investigates the complexity of pain mechanisms involved in postconcussion syndrome. Sex-specific research expanding on therapeutic targets such as sleep disorders and psychosocial distress holds the potential to assist in minimizing the suffering of persons with mTBI.

## Acknowledgments

The authors thank the 2013/2015 Frederick Banting and Charles Best Doctoral Research Award from the Canadian Institutes of Health Research and the 2015/2016 postdoctoral fellowship from the Acquired Brain Injury Laboratory of the Rehabilitation Sciences Institute at the University of Toronto and the Toronto Rehabilitation Institute-University Health Network research fellowship (to TM); and the Canadian Institutes for Health Research Grant–Institute for Gender and Health (#CGW-126580) (to AC) for the support.

## Supplementary Material

Supplemental Digital Content
