# Supplementary material for: Concussion/mild traumatic brain injury-related chronic pain in males and females: A diagnostic modelling study
Source: Medicine (Baltimore). 2017 Feb 17;96(7):e5917. doi: 10.1097/MD.0000000000005917 (PMC5319497; doi:10.1097/MD.0000000000005917)
Supplement: Supplemental Digital Content [file medi-96-e5917-s001.doc]

Supplementary Table 1. Categories and types of variables collected.

| Category | Type | Variable (standardized measure used, where applicable) |
| --- | --- | --- |
| Physiological and brain injury-related | Continuous | Time since injury (TSI) |
|  | Categorical | Mechanism of injury |
|  | Binary | Loss of consciousness (LOC) |
|  |  | Post-traumatic amnesia (PTA) |
|  |  | Previous head trauma |
|  |  | Hematoma, lacerations associated with head injury |
|  |  | Head and neck neuroimaging data ( i.e., MRI/CT findings) |
| Social and cultural | Continuous | Age (years) |
|  |  | Weekly salary at injury |
|  | Categorical | Occupational category at injury |
|  | Binary | Sex |
|  |  | Dependent children in the household |
|  |  | Education |
|  |  | Marital status |
|  |  | Born in Canada |
|  |  | English first language |
|  |  | Working>40 hours at injury time |
| Medication/substance effect | Continuous | Number of cups of caffeinated drinks per day |
|  |  | Number of prescribed medications |
|  | Binary | Use of tricyclic antidepressants (TCAs) |
|  |  | Use of benzodiazepines (BZs) |
|  |  | Use of selective serotonin reuptake inhibitors (SSRIs) |
|  |  | Use of serotonin–norepinephrine reuptake inhibitors (SNRIs) |
|  |  | Use of β-blockers |
|  |  | Use of narcotic analgesics |

| Psychological, physical and medical | Continuous | Depression (PHQ-9) |
| --- | --- | --- |
|  |  | Anxiety (HADS-A) |
|  |  | Insomnia (ISI) |
|  |  | Total number of comorbid disorders |
|  | Binary | DSM-IV TR disorders (mood, anxiety, adjustment, substance-related, somatoform, malingering, sleep, and cognitive disorders) |
|  |  | DSM-IV-TR personality traits |
|  |  | Diagnosed disorders (heart disease, arthritis, diabetes mellitus, thyroid dysfunction (hypo- or hyperthyroidism) |
|  |  | Symptom load impacting functioning |
| Behavioural | Continuous | Sleep-related breathing disorder risk factors (STOP-Bang) |
|  |  | Alcohol intake, daily (portion(s) of beer, wine, or liquor) |
|  |  | Coffee/Tea, cups a day |
|  |  | Number of prescribed medications |
|  |  | Body mass index |
|  |  | Total sleep time (STQ) |
|  | Binary | DSM-IV-TR possible/probable malingering |
|  |  | Current working status |
|  |  | Family difficulties |
|  |  | Tension with employer |
|  |  | Tension with WSIB |
|  |  | Weight gain since injury |
|  |  | Napping (SNS) |
|  | Categorical | Bedtime stability (STQ) |
|  |  | Wake-up time stability (STQ) |

Acronyms used : Patient Health Questionnaire-9 (PHQ-9); Hospital Anxiety and Depression Scale (HADS-A); Insomnia Severity Index (ISI); Sleep timing questionnaire (STQ); Insomnia severity index (ISI); Restless legs questionnaire (RLQ); Swiss narcolepsy scale (SNS); Workers’ Safety and Insurance Board (WSIB)

Supplementary Table 2 (2.1.). Results of the final regression analyses for the total sample (males and females combined) with the sleep item removed from the depression measure; age- and sex-adjusted.

| Variable | Parameter Estimate | Standard Error | Type II SS | F Value | Pr > F |
| --- | --- | --- | --- | --- | --- |
| Intercept | 9.69468 | 2.09044 | 548.29137 | 21.51 | <.0001 |
| Depression | 0.28091 | 0.10711 | 175.33253 | 6.88 | 0.0104 |
| Time since injury | 0.00083108 | 0.00048270 | 75.56891 | 2.96 | 0.0888 |
| Insomnia | 0.20315 | 0.10488 | 95.65246 | 3.75 | 0.0561 |
| Struck by another person | -3.92482 | 1.73217 | 130.88146 | 5.13 | 0.0260 |
| Fall from elevation | 2.68969 | 1.43082 | 90.08524 | 3.53 | 0.0636 |
| Tension with insurer | 4.46303 | 1.50015 | 225.63787 | 8.85 | 0.0038 |
| English first language | -3.15895 | 1.37007 | 135.52545 | 5.32 | 0.0236 |

| Step | Variable Entered | Number Vars In | Partial R-Square | Model R-Square | C(p) | F Value | Pr > F |
| --- | --- | --- | --- | --- | --- | --- | --- |
| 1 | Depression | 1 | 0.1663 | 0.1663 | 33.1974 | 17.95 | <.0001 |
| 2 | Mechanism of injury struck by another person | 2 | 0.0648 | 0.2311 | 25.7789 | 7.50 | 0.0075 |
| 3 | Tension with insurer | 3 | 0.0438 | 0.2749 | 21.4159 | 5.31 | 0.0235 |
| 4 | Insomnia | 4 | 0.0474 | 0.3222 | 16.5267 | 6.08 | 0.0156 |
| 5 | English first language | 5 | 0.0371 | 0.3593 | 13.1338 | 4.98 | 0.0282 |
| 6 | Mechanism of injury fall from elevation | 6 | 0.0273 | 0.3867 | 11.1594 | 3.79 | 0.0549 |
| 7 | Time since injury | 7 | 0.0209 | 0.4076 | 10.1203 | 2.96 | 0.0888 |

Supplementary Table 2 (2.2). Results of the final regression analyses for male participants with the sleep item removed from the depression measure; age-adjusted.

| Variable | Parameter Estimate | Standard Error | Type II SS | F Value | Pr > F |
| --- | --- | --- | --- | --- | --- |
| Intercept | 1.55081 | 3.47614 | 2.97018 | 0.20 | 0.6567 |
| Total number of comorbid disorders | 0.76009 | 0.51374 | 32.66680 | 2.19 | 0.1457 |
| Anxiety | 0.45110 | 0.13064 | 177.93677 | 11.92 | 0.0012 |
| Insomnia | 0.35135 | 0.08862 | 234.59890 | 15.72 | 0.0002 |
| Fall from elevation | 2.75307 | 1.39914 | 57.77927 | 3.87 | 0.0550 |
| Exposure to explosion | 6.88698 | 2.88151 | 85.24638 | 5.71 | 0.0209 |
| Tension with insurer | 3.28736 | 1.49788 | 71.87903 | 4.82 | 0.0332 |
| English first language | -2.96683 | 1.40675 | 66.375443 | 4.45 | 0.0403 |
| Currently working | -3.32150 | 1.17597 | 119.05213 | 7.98 | 0.0069 |
| Working >40 hours/week | 4.41904 | 2.42467 | 49.56895 | 3.32 | 0.0747 |

| Step | Variable Entered | Variable Removed | Number Vars In | Partial R-Square | Model R-Square | C(p) | F Value | Pr > F |
| --- | --- | --- | --- | --- | --- | --- | --- | --- |
| 1 | Depression |  | 1 | 0.2465 | 0.2465 | 46.5100 | 17.99 | <.0001 |
| 2 | Fall from elevation |  | 2 | 0.1358 | 0.3823 | 30.5706 | 11.88 | 0.0011 |
| 3 | Anxiety |  | 3 | 0.0532 | 0.4356 | 25.5388 | 5.00 | 0.0296 |
| 4 | English first language |  | 4 | 0.0572 | 0.4928 | 19.9838 | 5.86 | 0.0190 |
| 5 | Currently working |  | 5 | 0.0474 | 0.5402 | 15.7295 | 5.25 | 0.0261 |
| 6 | Insomnia |  | 6 | 0.0357 | 0.5758 | 13.0189 | 4.20 | 0.0456 |
| 7 | Working >40 hours/week |  | 7 | 0.0332 | 0.6090 | 10.6407 | 4.15 | 0.0469 |
| 8 |  | Depression | 6 | 0.0220 | 0.5870 | 11.5489 | 2.76 | 0.1031 |
| 9 | Exposure to explosion |  | 7 | 0.0337 | 0.6207 | 9.0951 | 4.36 | 0.0421 |
| 10 | Tension with insurer |  | 8 | 0.0338 | 0.6545 | 6.6254 | 4.70 | 0.0351 |
| 9 | Total number of comorbid disorders |  | 9 | 0.0154 | 0.6699 | 6.5950 | 2.19 | 0.1457 |
| 10 |  | Total number of comorbid disorders | 8 | 0.0154 | 0.6545 | 6.6254 | 2.19 | 0.1457 |

Supplementary Table 2 (2.3). Results of the final regression analyses for female participants with the sleep item removed from the depression measure; age-adjusted.

| Variable | Parameter Estimate | Standard Error | Type II SS | F Value | Pr > F |
| --- | --- | --- | --- | --- | --- |
| Intercept | 19.70704 | 2.75642 | 1080.68310 | 51.12 | <.0001 |
| Daytime sleepiness | -0.50031 | 0.15804 | 211.88013 | 10.02 | 0.0039 |
| Education >than high school | -4.40480 | 2.02852 | 99.68729 | 4.72 | 0.0392 |
| Stop-Bang (sleep apnea) | 1.74974 | 0.58935 | 186.35370 | 8.81 | 0.0063 |
| Working> 40 hours/week | -4.35732 | 1.74709 | 131.50889 | 6.22 | 0.0193 |

| Step | Variable Entered | Number Vars In | Partial R-Square | Model R-Square | C(p) | F Value | Pr > F |
| --- | --- | --- | --- | --- | --- | --- | --- |
| 1 | Education > than high school | 1 | 0.1799 | 0.1799 | 14.4193 | 6.36 | 0.0174 |
| 2 | Daytime sleepiness | 2 | 0.1079 | 0.2878 | 10.9693 | 4.24 | 0.0488 |
| 3 | Stop-Bang (sleep apnea) | 3 | 0.1338 | 0.4217 | 6.2103 | 6.25 | 0.0188 |
| 4 | Working > 40 hours/week | 4 | 0.1117 | 0.5333 | 2.5711 | 6.22 | 0.0193 |
